# Construction and validation of a predictive model for exclusive breastfeeding at discharge based on the information-motivation-behavioral skills theory

**DOI:** 10.3389/fmed.2025.1683293

**Published:** 2025-10-30

**Authors:** Na Yin, Shanshan Shan, Jie Bai, Hongxia Lu, Yangyang Wang, Jiaqi Li, Hui Jiang, Ju Zhang

**Affiliations:** 1 College of Nursing, Hangzhou Normal University, Hangzhou, China; 2 Nursing Department, Shanghai First Maternity and Infant Hospital, Obstetrics and Gynecology Hospital Afffliated to Tongji University School of Medicine, Shanghai, China; 3 Affiliated Maternal and Child Health Hospital of East China Normal University, Shanghai Changning District Maternal and Child Health Hospital, Shanghai, China

**Keywords:** information–motivation–behavioral skills (IMB) model, exclusive breastfeeding, risk prediction, nomogram, predictive model

## Abstract

**Background:**

Despite the well-established health benefits of exclusive breastfeeding for both mothers and infants, breastfeeding rates in China remain suboptimal. This study, guided by the Information–Motivation–Behavioral Skills (IMB) model, aimed to develop and validate a predictive model for exclusive breastfeeding at discharge to facilitate the early identification of high-risk mothers and enable timely clinical interventions.

**Methods:**

In this prospective observational study conducted from February to June 2025, a total of 623 postpartum women were recruited, with 592 meeting the inclusion criteria. Of these, 448 were allocated to the model development group, while 144 from a different hospital formed the external validation group. Demographic and breastfeeding-related variables were collected via questionnaires and electronic medical records. Logistic regression was employed to identify significant predictors and construct a nomogram. Model performance was evaluated using the area under the receiver operating characteristic curve (AUC), calibration plots, the Hosmer–Lemeshow goodness-of-fit test, and decision curve analysis (DCA), and externally validated using an independent cohort.

**Results:**

Both univariate and multivariate logistic regression analyses identified newborn sex, early skin-to-skin contact, breastfeeding attitude, breastfeeding self-efficacy, and LATCH score as significant predictors of exclusive breastfeeding at discharge. The nomogram exhibited good discriminatory ability, with an AUC of 0.76 (95% CI, 0.70–0.81) in the development group and 0.66 (95% CI, 0.56–0.75) in the validation group. The Hosmer–Lemeshow test indicated good model calibration (*p* > 0.05), and decision curve analysis demonstrated favorable clinical applicability.

**Conclusion:**

This study successfully constructed and preliminarily validated a pure breastfeeding prediction model based on the IMB theory. The model demonstrates good calibration and moderate discriminatory ability, enabling clinicians to identify mothers at higher risk for exclusive breastfeeding failure early before discharge. Although its external validation performance suggests that its generalizability requires further validation in larger samples and more centers, its robust theoretical foundation positions it as a valuable risk assessment and screening tool. This provides a meaningful reference framework and methodological starting point for developing precise, efficient, and targeted nursing interventions in the future.

## 1 Introduction

Breastfeeding is the most natural and optimal form of infant nutrition, offering a unique blend of nutrients and bioactive compounds. It is widely acknowledged as the gold standard for infant feeding (1). Between 1990 and 2021, China’s under-five mortality rate declined dramatically, from 53.6 to 6.9 deaths per 1,000 live births (2). Often referred to as the infant’s “first vaccine,” breast milk is rich in antibacterial and anti-inflammatory components—including Bifidobacterium, lactoferrin, and secretory immunoglobulin A (sIgA)—and is estimated to prevent approximately 600,000 child deaths annually due to diarrhea and pneumonia. Breastfeeding also confers long-term health benefits for mothers, including a reduced risk of breast cancer, ovarian cancer, and a range of non-communicable diseases (3, 4). Beyond fulfilling infants’ nutritional requirements, breastfeeding enhances the emotional bond between mother and infant through skin-to-skin contact (5).

The World Health Organization (WHO) recommends initiating breastfeeding within the first hour of birth, exclusively breastfeeding for the first 6 months of life, and continuing breastfeeding alongside complementary feeding up to 2 years of age or longer. However, despite most mothers initiating breastfeeding shortly after delivery, the global prevalence of exclusive breastfeeding remains suboptimal. According to a UNICEF survey, the global exclusive breastfeeding rate at 6 months of age is only 41% (6). Notably, substantial regional disparities exist, with China’s exclusive breastfeeding rate reported at just 29% (7).

Breastfeeding is shaped by a complex interplay of individual, environmental, and structural factors, with adverse influences at any level potentially leading to early discontinuation (8). For example, inadequate breastfeeding knowledge and skills, low maternal self-efficacy, unfavorable nipple conditions (e.g., flat, inverted, cracked, or painful), and sociodemographic factors such as maternal age, occupation, education level, and household income can all negatively impact breastfeeding practices. Evidence suggests that newborns who are exclusively breastfed at discharge are less likely to experience early cessation of breastfeeding and exhibit better long-term health outcomes (9). In addition, the perinatal hospital stay plays a critical role in initiating and sustaining breastfeeding between mother and infant (10). Compared with managing breastfeeding challenges postpartum, early identification of risk factors and the implementation of preventive strategies are more effective.

This study draws on the Information–Motivation–Behavioral Skills (IMB) model, which posits that individuals are more likely to adopt a specific behavior when both adequate information and sufficient motivation are present (11). According to the model, information refers to a mother’s knowledge of breastfeeding, while motivation encompasses her attitudes, breastfeeding self-efficacy, and perceived social support. Behavioral skills emphasize the practical techniques and competencies required for mothers to initiate and sustain effective breastfeeding.

Risk prediction models employ data mining techniques to quantitatively assess the likelihood of future events. By identifying variables with strong predictive value for exclusive breastfeeding, these models provide a scientific basis for personalized interventions. In recent years, such models have been widely applied to clinical event prediction and have demonstrated robust predictive performance in numerous studies (12). This study aimed to develop and validate a predictive model for exclusive breastfeeding at discharge, grounded in the IMB framework. The model facilitates early identification of risk factors influencing breastfeeding success, supports precise interventions, and holds significant potential for improving breastfeeding rates and extending breastfeeding duration.

## 2 Materials and methods

### 2.1 Design and participants

This prospective observational study was conducted from February to July 2025 in the obstetric wards of two specialized maternal and child health hospitals in Shanghai. One hospital reported over 25,000 deliveries annually, and the other exceeded 10,000. Participants were mainly recruited from Shanghai, Anhui, Zhejiang, Jiangsu, Henan, and Hebei provinces, representing a broad geographical distribution across China. Post-delivery breastfeeding education formed part of routine ward care at both hospitals. The first bedside counseling was provided at the first breastfeeding attempt (i.e., immediately after birth during early skin-to-skin contact when feasible, or once clinically stable after cesarean), and a reinforcement session was delivered prior to discharge. Trained maternity nurses/lactation staff provided the counseling, with brief bedside clarifications available during routine nursing rounds. Inclusion criteria were as follows: women with a prenatal intention to breastfeed who met all of the following conditions: (1) aged 18 years or older; (2) singleton, full-term pregnancy (gestational age ≥37 weeks); (3) sufficient reading, communication, and comprehension skills to independently complete the questionnaire; and (4) provided informed consent and voluntarily participated in the study. Exclusion criteria were: (1) contraindications to breastfeeding, such as use of specific medications, a history of breast surgery, acute or chronic infectious diseases, or congenital anomalies in the newborn; (2) diagnosed mental illness; and (3) communication impairments.

### 2.2 Sample selection

#### 2.2.1 Sample size

According to the sample size estimation formula for logistic regression—n = number of predictors × (5 ~ 10) ÷ event rate—a total of 28 predictors and an exclusive breastfeeding rate of 80.3% at discharge indicated that the modeling group required at least 209 to 418 participants, accounting for a 20% potential invalid response rate. A total of 448 participants were ultimately enrolled in the modeling group, indicating adequate statistical power for the analyses.

#### 2.2.2 External validation cohort

Women who gave birth between May and July 2025 at a secondary-level specialized hospital in Changning District, Shanghai, were included in the external validation cohort. The inclusion and exclusion criteria matched those of the modeling group. A total of 144 participants were included for external validation of the predictive model.

### 2.3 Data collection and data sources

Data for this study were collected through a combination of paper-based questionnaires and the hospital’s electronic medical record system. First, after obtaining informed consent, trained researchers explained the study objectives and instructions for completing the questionnaire, and distributed paper-based forms during participants’ hospital stay. The questionnaire captured information on exclusive breastfeeding at discharge, duration of maternity leave, per capita monthly household income, breastfeeding attitudes, and maternal self-efficacy. Completed questionnaires were collected on site and checked for completeness and accuracy. Additionally, general demographic and clinical information of mothers and newborns was extracted from the hospital information system (HIS), including maternal age, parity, mode of conception, and mode of delivery. The final dataset comprised the following variables:

The primary outcome variable was exclusive breastfeeding at discharge. As defined by the World Health Organization, exclusive breastfeeding means feeding infants solely with breast milk, excluding all other liquids or solids—not even water—except for oral rehydration solutions, vitamins, minerals, and medications. This definition was clearly explained in the questionnaire.

Independent variables included the following:

Maternal and neonatal factors: maternal age, education level, parity, mode of conception, annual household income, maternity leave duration, previous breastfeeding experience, birth plan, mode of delivery, history of cesarean section, pre-pregnancy BMI, analgesia method, episiotomy, family support, midwifery clinic attendance, breastfeeding education participation, perceived insufficient milk supply, intended duration of breastfeeding, infant sex, timing of first breastfeeding, and whether skin-to-skin contact occurred.

In addition, the following validated instruments were used to assess breastfeeding-related variables:

1. This study used the breastfeeding knowledge scale designed by Zhao Min (13), comprises 17 items—11 on the benefits of breastfeeding and 6 on breastfeeding skills. Responses are scored as “Yes,” “No,” or “Uncertain,” with 1 point awarded for each correct answer. Total scores range from 0 to 17, with higher scores reflecting greater breastfeeding knowledge. The Cronbach’s *α* coefficient was 0.82.
2. The Iowa Infant Feeding Attitude Scale (IIFAS), developed by De la Mora et al. (14), evaluates maternal attitudes toward infant feeding and serves as a theoretical foundation for breastfeeding education. The scale contains 17 items rated on a 5-point Likert scale (1 = strongly disagree, 5 = strongly agree), yielding a total score between 17 and 85. Lower scores indicate a preference for formula feeding, whereas higher scores indicate a preference for breastfeeding. Reported Cronbach’s *α* coefficients range from 0.50 to 0.86 across different countries and populations.
3. The Breastfeeding Self-Efficacy Scale–Short Form (BSES-SF), developed by Dennis (15), assesses maternal self-efficacy during breastfeeding. It includes 14 items scored on a 5-point Likert scale, with a maximum total score of 70. Higher scores represent greater breastfeeding self-efficacy. The Cronbach’s *α* coefficient was 0.94.
4. The LATCH Breastfeeding Assessment Tool, developed by Jensen et al. (16), evaluates the overall breastfeeding process by assessing five domains: L (Latch), A (Audible swallowing), T (Type of nipple), C (Comfort), and H (Hold/help). Each domain is scored on a scale of 0 to 2, resulting in a maximum total score of 10. Higher scores indicate a greater likelihood of successful breastfeeding. The reported Cronbach’s *α* coefficient was 0.93.

### 2.4 Statistical analysis

**Figure 1:**
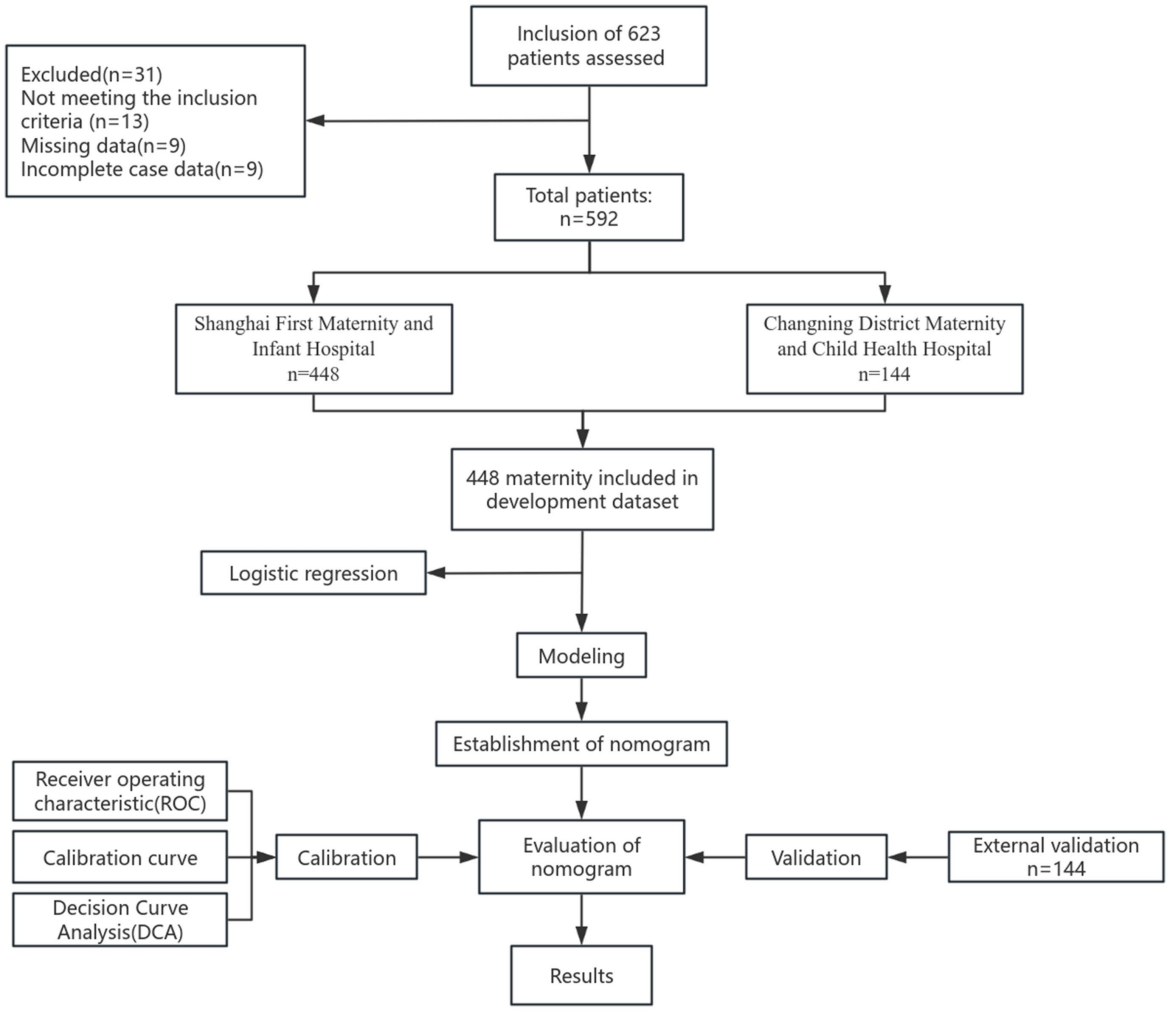
Flow chart of the study.

Continuous variables with a normal distribution were presented as mean ± standard deviation (SD), while non-normally distributed variables were described using median and interquartile range (IQR). Categorical variables were summarized as counts (*n*) and proportions (%). Group comparisons were conducted using the chi-square (χ^2^) test or Fisher’s exact test, as appropriate. In the training cohort, univariate logistic regression analysis was initially performed to identify potential risk factors associated with exclusive breastfeeding. Variables with a *p*-value < 0.1 were subsequently entered into a multivariate logistic regression model. Backward stepwise selection based on the Wald statistic was employed to determine the optimal subset of predictors. A nomogram was constructed using R software (version 4.5.0). Model calibration was assessed using the Hosmer–Lemeshow goodness-of-fit test. Predictive performance was evaluated by calculating the area under the receiver operating characteristic curve (AUC). Sensitivity and specificity were used to quantify diagnostic accuracy, and decision curve analysis (DCA) was performed to evaluate clinical utility. External validation was conducted using an independent cohort from a geographically distinct population. A two-sided *p*-value < 0.05 was considered statistically significant. All statistical analyses were performed using R software (version 4.5.0). The overall study procedure is illustrated in Figure 1.

## 3 Results

### 3.1 Training set and validation set maternal characteristics

A total of 623 postpartum women were initially enrolled. After excluding 13 participants who did not meet the inclusion criteria and 18 with missing or incomplete data, 592 participants were included in the final analysis. Of these, 448 were allocated to the training cohort and 144 to the external validation cohort. The mean age across the entire sample was 31.97 ± 3.44 years. In the training cohort, 360 women practiced exclusive breastfeeding, and 88 used mixed or formula feeding, yielding an exclusive breastfeeding rate of 80.4%. In the validation cohort, 101 women exclusively breastfed and 43 used mixed or formula feeding, resulting in a rate of 70.1%. Baseline characteristics revealed differences between the training and validation cohorts in certain demographic and clinical variables, as detailed in Table 1.

**Table 1:** Training set and validation set maternal characteristics.

Variables	Total (*n* = 592)	Training set (*n* = 448)	External validation set (*n* = 144)	*P*
Age, mean ± SD	31.97 ± 3.44	31.97 ± 3.53	31.98 ± 3.15	0.975
Education level, *n*(%)				0.003
Junior high or below	9 (1.52)	6 (1.34)	3 (2.08)	
High school or vocational school	12 (2.03)	8 (1.79)	4 (2.78)	
College or bachelor’s degree	407 (68.75)	294 (65.62)	113 (78.47)	
Graduate student	164 (27.70)	140 (31.25)	24 (16.67)	
Household registration, *n*(%)				0.318
Local Shanghai	133 (22.47)	105 (23.44)	28 (19.44)	
Outside Shanghai	459 (77.53)	343 (76.56)	116 (80.56)	
Number of children, *n*(%)				0.222
1 Child	470 (79.39)	363 (81.03)	107 (74.31)	
2 Children	112 (18.92)	78 (17.41)	34 (23.61)	
3 or more children	10 (1.69)	7 (1.56)	3 (2.08)	
Mode of conception, *n*(%)				0.018
Natural conception	538 (90.88)	400 (89.29)	138 (95.83)	
Assisted conception	54 (9.12)	48 (10.71)	6 (4.17)	
Pre-delivery BMI, *n*(%)				0.057
Underweight (<18.49 kg/m^2^)	59 (9.97)	49 (10.94)	10 (6.94)	
Normal (18.5 ~ 24.99 kg/m^2^)	470 (79.39)	345 (77.01)	125 (86.81)	
Overweight (25 ~ 29.99 kg/m^2^)	55 (9.29)	46 (10.27)	9 (6.25)	
Obese(≥30 kg/m^2^)	8 (1.35)	8 (1.79)	0 (0.00)	
Mode of delivery, *n*(%)				0.007
Vaginal delivery	313 (52.87)	233 (52.01)	80 (55.56)	
Cesarean delivery	257 (43.41)	204 (45.54)	53 (36.81)	
Forceps-assisted delivery	22 (3.72)	11 (2.46)	11 (7.64)	
History of cesarean section, *n*(%)				0.363
Yes	47 (7.94)	33 (7.37)	14 (9.72)	
None	545 (92.06)	415 (92.63)	130 (90.28)	
Pain relief method, *n*(%)				0.066
Epidural anesthesia	307 (51.86)	221 (49.33)	86 (59.72)	
Spinal anesthesia	262 (44.26)	207 (46.21)	55 (38.19)	
None	23 (3.89)	20 (4.46)	3 (2.08)	
Delivery companion, *n*(%)				0.004
Yes	313 (52.87)	222 (49.55)	91 (63.19)	
No	279 (47.13)	226 (50.45)	53 (36.81)	
Newborn gender, *n*(%)				0.494
Female	286 (48.31)	220 (49.11)	66 (45.83)	
Male	306 (51.69)	228 (50.89)	78 (54.17)	
Mother-infant separation, *n*(%)				<0.001
Yes	111 (18.75)	106 (23.66)	5 (3.47)	
No	481 (81.25)	342 (76.34)	139 (96.53)	
Received breastfeeding education, *n*(%)				<0.001
Yes	498 (84.12)	354 (79.02)	144 (100.00)	
No	94 (15.88)	94 (20.98)	0 (0.00)	
Exclusive breastfeeding at discharge, *n*(%)				0.010
Yes	461 (77.87)	360 (80.36)	101 (70.14)	
No	131 (22.13)	88 (19.64)	43 (29.86)	
Breastfeeding experience, *n*(%)				0.162
Yes	139 (23.48)	99 (22.10)	40 (27.78)	
No	453 (76.52)	349 (77.90)	104 (72.22)	
Perceived insufficient milk, *n*(%)				0.764
Yes	372 (62.84)	280 (62.50)	92 (63.89)	
No	220 (37.16)	168 (37.50)	52 (36.11)	
Planned pregnancy and delivery, *n*(%)				0.481
Yes	516 (87.16)	394 (87.95)	122 (84.72)	
No	75 (12.84)	53 (12.05)	22 (15.28)	
First breastfeeding after delivery, *n*(%)				<0.001
Within 1 h of delivery	131 (22.13)	68 (15.18)	63 (43.75)	
Within 24 h of delivery	430 (72.64)	358 (79.91)	72 (50.00)	
After 1 day of delivery	31 (5.24)	22 (4.91)	9 (6.25)	
Skin-to-skin contact, *n*(%)				<0.001
Yes	519 (87.67)	425 (94.87)	94 (65.28)	
No	73 (12.33)	23 (5.13)	50 (34.72)	
Maternity leave, *n*(%)				0.261
<3 months	9 (1.52)	8 (1.79)	1 (0.69)	
3 ~ 4 months	16 (2.70)	9 (2.01)	7 (4.86)	
4 ~ 6 months	500 (84.46)	381 (85.04)	119 (82.64)	
≥6 months	67 (11.32)	50 (11.16)	17 (11.81)	
Family monthly income, *n*(%)				0.702
<5,000 RMB	7 (1.18)	6 (1.34)	1 (0.69)	
5,000 ~ 10,000 RMB	137 (23.14)	106 (23.66)	31 (21.53)	
>10,000 RMB	448 (75.68)	336 (75.00)	112 (77.78)	
Breastfeeding duration expectation, *n*(%)				0.389
1 ~ 3 months	35 (5.91)	27 (6.03)	8 (5.56)	
4 ~ 6 months	330 (55.74)	258 (57.59)	72 (50.00)	
7 ~ 12 months	216 (36.49)	155 (34.60)	61 (42.36)	
13 ~ 24 months	11 (1.86)	8 (1.79)	3 (2.08)	
Family support level, *n*(%)				0.507
Low	5 (0.84)	2 (0.67)	2 (1.39)	
Medium	103 (17.40)	76 (16.96)	27 (18.75)	
High	484 (81.76)	369 (82.37)	115 (79.86)	
Complications, mean ± SD				<0.001
Present	155 (26.18)	96 (21.43)	59 (40.97)	
None	437 (73.82)	352 (78.57)	85 (59.03)	
BKQ, mean ± SD	12.02 ± 2.94	11.77 ± 2.99	12.80 ± 2.65	<0.001
IIFAS, mean ± SD	56.99 ± 6.36	57.02 ± 6.44	56.92 ± 6.10	0.871
BSES-SF, mean ± SD	46.71 ± 10.51	46.42 ± 10.66	47.63 ± 10.02	0.228
LATCH, mean ± SD	8.17 ± 1.22	8.18 ± 1.13	8.12 ± 1.47	0.620

### 3.2 Model development

Univariate logistic regression analysis in the training cohort revealed that neonatal sex, early skin-to-skin contact, duration of maternity leave, intended breastfeeding duration, maternal attitude toward feeding, breastfeeding self-efficacy, and LATCH score were significantly associated with exclusive breastfeeding at discharge (*p* < 0.05), as detailed in Supplementary material 1. Variables with *p* < 0.05 in the univariate analysis were subsequently included in a multivariate logistic regression model. The analysis identified neonatal sex, skin-to-skin contact, maternal attitude toward feeding, breastfeeding self-efficacy, and LATCH score as independent predictors of exclusive breastfeeding at discharge (see Table 2).

**Table 2:** Multivariate logistic analysis results for exclusive breastfeeding at discharge.

Variables	β	S.E.	Z	*P*	OR (95%CI)
Newborn gender					
Female					1.00 (Reference)
Male	−0.60	0.26	−2.26	0.024	0.55 (0.33 ~ 0.92)
Skin-to-skin contact					
No					1.00 (Reference)
Yes	1.41	0.46	3.07	0.002	4.11 (1.66 ~ 10.15)
IIFAS	0.07	0.03	2.56	0.010	1.07 (1.02 ~ 1.12)
BSES-SF	0.04	0.02	2.67	0.008	1.04 (1.01 ~ 1.07)
LATCH	0.34	0.11	3.22	0.001	1.41 (1.14 ~ 1.74)

### 3.3 Establish a risk prediction model

**Figure 2:**
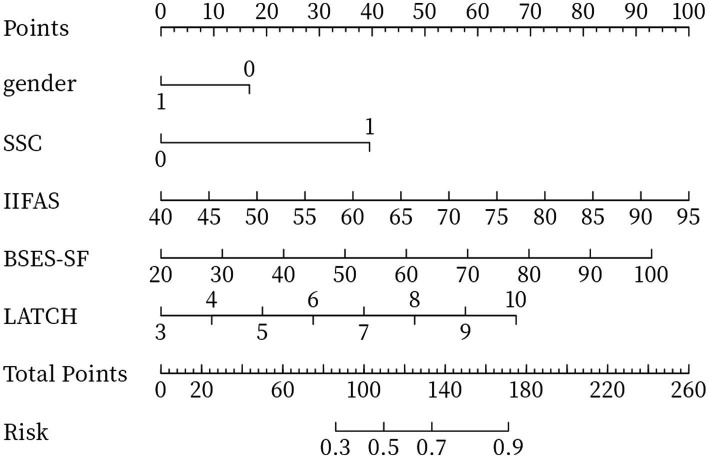
Nomogram for exclusive breastfeeding at discharge.

Based on multivariate logistic regression analysis, five significant predictors were incorporated into a predictive model (Figure 2), from which a personalized nomogram was developed to estimate the likelihood of exclusive breastfeeding at discharge. The nomogram is applied by assigning a score to each predictive variable according to its position on the chart, then summing these scores to generate a total score.

### 3.4 Evaluation of predictive models

**Figure 3:**
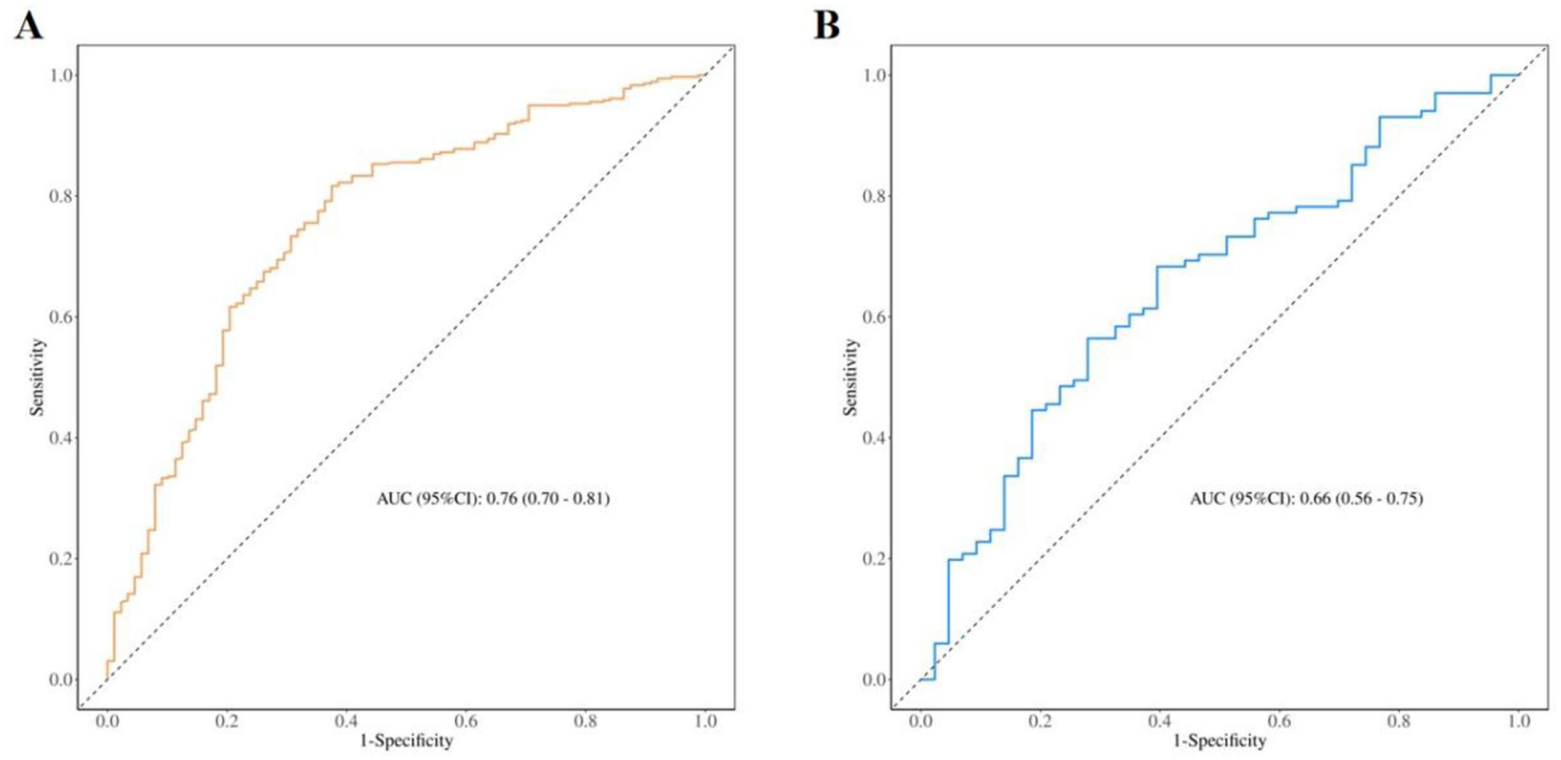
ROC curves for the training set **(A)** and validation set **(B)**.

**Figure 4:**
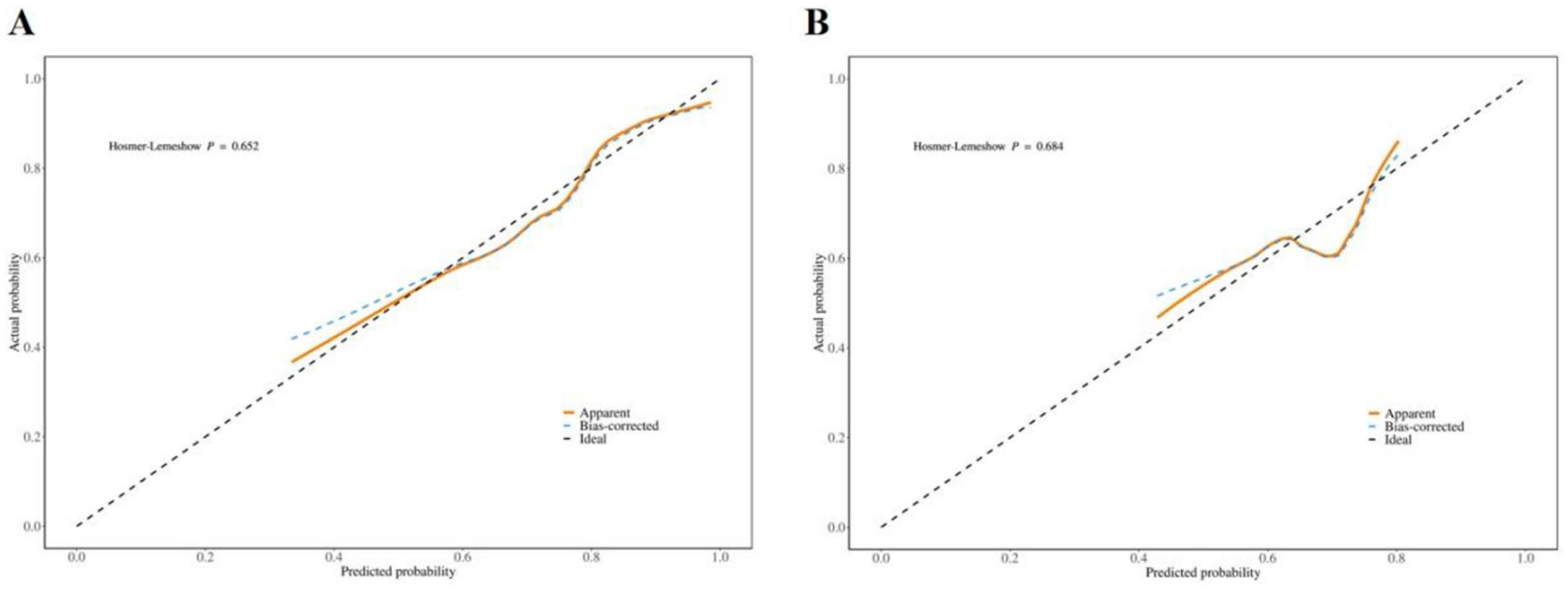
Calibration curves of training set **(A)** and validation set **(B)** line charts.

**Figure 5:**
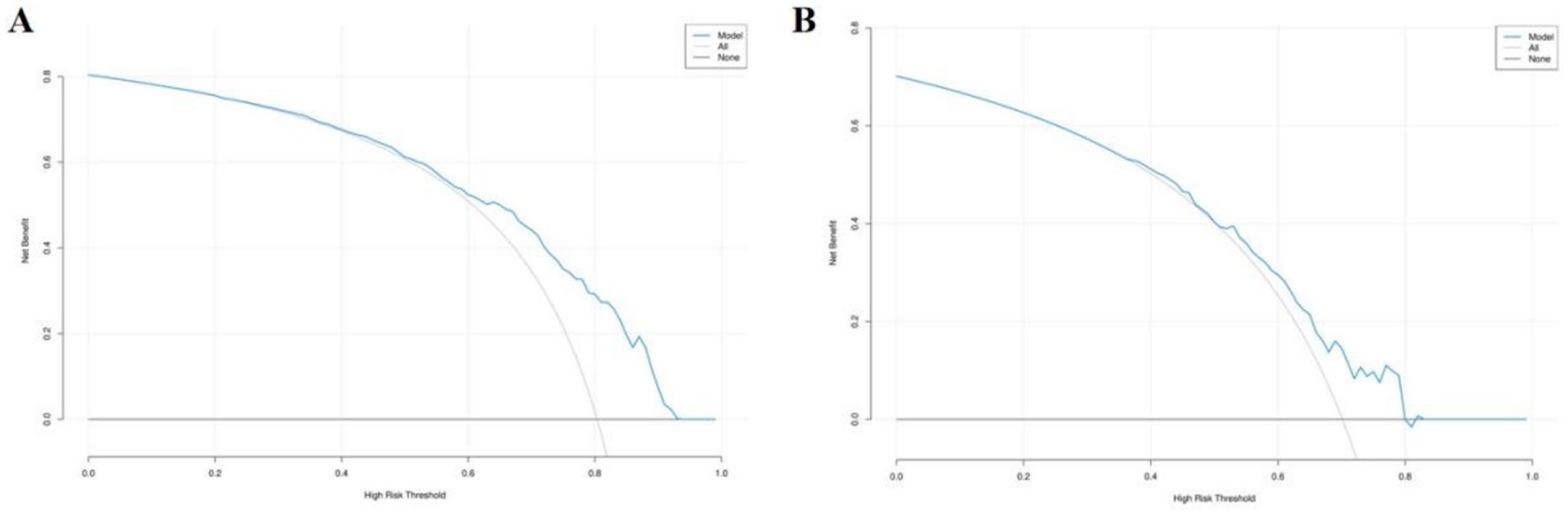
DCA curves for training set **(A)** and validation set **(B)** scatter plots.

The model’s discriminative performance was evaluated using the area under the receiver operating characteristic (ROC) curve (AUC). The AUC was 0.76 (95% CI: 0.70–0.81) for the training set and 0.66 (95% CI: 0.56–0.75) for the validation set (Figures 3A,B). The Hosmer–Lemeshow goodness-of-fit test indicated that the model exhibited good calibration in both the training cohort (χ^2^ = 6.23, *p* = 0.62) and the validation cohort (χ^2^ = 7.28, *p* = 0.51). As illustrated in Figure 4, the model demonstrated robust predictive performance in both the training and validation cohorts, with consistent calibration across a range of probability thresholds following bias correction. Decision curve analysis (DCA), shown in Figure 5, indicated that the model offered an acceptable net benefit and predictive utility.

To further evaluate the incremental value of the additional variables, we constructed a simplified model including only the LATCH score, the Breastfeeding Self-Efficacy Scale (BSES), and the Iowa Infant Feeding Attitude Scale (IIFAS), and compared it with the full model that additionally incorporated neonatal sex and early skin-to-skin contact. The simplified model yielded an AUC of 0.73, whereas the full model achieved an AUC of 0.76. The likelihood ratio test demonstrated that the full model provided a significantly better fit than the simplified model (χ^2^ = 16.87, df = 2, *p* = 0.0002), indicating that the additional variables contributed independent and statistically significant predictive information.

## 4 Discussion

Guided by the IMB theory, this study developed a predictive model for exclusive breastfeeding at discharge using multivariate logistic regression. The model was visualized as a nomogram and externally validated. This study prospectively identified key factors associated with exclusive breastfeeding at discharge. The findings indicated that neonatal sex, early skin-to-skin contact, maternal feeding attitude, breastfeeding self-efficacy, and LATCH score were significant predictors of exclusive breastfeeding at discharge. The primary purpose of this predictive model is early risk screening. The nomogram not only provides an overall risk probability but also retains the individual score for each predictive factor, enabling clinicians to quickly identify specific weaknesses in breastfeeding skills, attitude, or self-efficacy and thereby implement individualized interventions.

Additionally, the predictive model developed in this study demonstrated good discrimination in the development cohort (AUC = 0.76); however, its predictive performance declined in the external validation cohort (AUC = 0.66). This discrepancy may be attributable to the fact that the validation cohort was derived from another hospital, where unmeasured differences in maternal demographic characteristics, breastfeeding support measures, and in-hospital management processes could have influenced the effect sizes of some predictive variables.

This study confirms that early skin-to-skin contact (SSC) significantly enhances the likelihood of exclusive breastfeeding at discharge, aligning with previous evidence that early maternal–infant contact promotes lactogenic hormone release, activates the sucking reflex, and fosters emotional bonding between mother and infant (17). SSC involves placing a naked newborn in a prone position on the mother’s bare chest, followed by drying, hatting, and covering both with a warm blanket (18). According to WHO guidelines, SSC should be maintained for at least 60 min. A cross-institutional survey in two Shanghai hospitals found SSC implementation rates of 94.9 and 65.3%, with corresponding exclusive breastfeeding rates at discharge of 80.4 and 70.1%, respectively, suggesting a positive correlation between SSC prevalence and exclusive breastfeeding outcomes. Evidence suggests that newborns who do not undergo SSC face a higher risk of hospital readmission within the first few days after birth, commonly due to jaundice and feeding difficulties (19). SSC plays a critical role in initiating breastfeeding early (20). A controlled clinical trial demonstrated that newborns exposed to SSC had a higher likelihood of successful first-hour feeding and were more likely to sustain exclusive breastfeeding up to 4 months postpartum (21). Moreover, SSC has been shown to alleviate maternal anxiety and facilitate the positive reconstruction of maternal identity (22). It is recommended that SSC be integrated into routine postpartum care. Enhancing delivery room protocols and staff training can improve awareness and implementation of its clinical benefits.

Maternal attitude toward breastfeeding emerged as a key predictor of exclusive breastfeeding at discharge in this study. Previous studies have identified maternal attitudes toward breastfeeding as strong predictors of a mother’s intention to breastfeed, as well as the initiation and continuation of breastfeeding practices (23, 24). Infant feeding decisions are frequently made during the antenatal period (23). Therefore, upon admission, obstetric nurses routinely inquire about pregnant women’s intended infant feeding method—exclusive breastfeeding, mixed feeding (breast milk and formula), or formula feeding. In our survey, the average maternal feeding attitude score was 57.0 ± 6.36, closely aligning with the IIFAS mean score (59.6 ± 7.3) reported by Abulreesh et al. in Saudi Arabia, but markedly lower than the scores observed in Spain (69.76 ± 7.75) and Jeddah, Saudi Arabia (81.39 ± 8.35), indicating a more positive breastfeeding attitude in those regions (25). These disparities may be influenced by cultural norms (26, 27), societal expectations, social networks (28), individual experiences (29), and the extent of breastfeeding education received (23). Evidence has shown that maternal feeding attitudes significantly influence a mother’s choice between breastfeeding and formula feeding (30). Mothers who hold positive attitudes toward breastfeeding are more likely to sustain breastfeeding and adopt practices conducive to optimal infant nutrition (31). Interventions aimed at improving maternal feeding attitudes should be strengthened during pregnancy and the early postpartum period, via antenatal classes and psychological support. The breastfeeding attitudes of healthcare professionals and midwives also exert a substantial influence on maternal behaviors, with their guidance playing a pivotal role in the initiation of breastfeeding (32).

This study identified maternal breastfeeding self-efficacy as a significant predictor of exclusive breastfeeding at discharge, in line with previous findings (33). Breastfeeding self-efficacy refers to a mother’s confidence in her ability to breastfeed, encompassing her perceived competence, effort, attitudes, and beliefs (34). Mothers with high breastfeeding self-efficacy are more capable of overcoming challenges, demonstrating stronger perseverance and adaptability, which in turn enhances the likelihood of exclusive breastfeeding (35). Studies suggest that mothers with prior breastfeeding experience tend to exhibit greater confidence, self-efficacy, intrinsic motivation, and a stronger intention to continue breastfeeding (36). Antenatal breastfeeding education has been shown to enhance maternal self-efficacy and subsequently improve exclusive breastfeeding rates at discharge (37).

The LATCH score, a standardized tool for evaluating breastfeeding interactions and maternal technique, has been shown to be positively associated with both the initiation and duration of exclusive breastfeeding. Higher LATCH scores indicate more effective infant latching, optimal maternal positioning, and greater maternal patience—factors that contribute to the establishment of a stable breastfeeding routine. Notably, several studies have reported a positive correlation between LATCH scores and sustained exclusive breastfeeding for up to 6 weeks postpartum (38, 39). Raghavan et al. (40) further observed that newborns with higher LATCH scores were significantly more likely to maintain exclusive breastfeeding at 6 weeks postpartum. The LATCH score may also serve as a predictive tool for early breastfeeding cessation, underscoring the importance of targeted support for mothers with low scores to sustain breastfeeding practices.

The gender of the newborn is an important factor influencing the rate of exclusive breastfeeding at discharge. This study found a significant negative correlation between male infants and lower rates of exclusive breastfeeding compared to female infants. These findings are consistent with research conducted in Kenya, Cameroon, Angola, and Ghana (41). Previous studies have indicated that female infants tend to breastfeed for a longer duration than their male counterparts (42). However, male infants may consume more breast milk daily than female infants (43). Consequently, some mothers may perceive that breast milk alone cannot meet the higher nutritional demands of male infants. Additionally, based on the belief that “male infants have a greater appetite,” they may introduce complementary foods earlier, which could affect the rate of exclusive breastfeeding (44).

Consistent with prior research, feeding type at discharge has been shown to significantly predict subsequent breastfeeding duration (45). demonstrated that mothers exclusively breastfeeding at discharge were substantially more likely to sustain EBF in the following weeks and months, compared with those who introduced formula supplementation during hospitalization (46). This evidence suggests that although our model only predicts EBF status at discharge, it nonetheless identifies a pivotal early stage that has downstream implications for breastfeeding continuation. Thus, the model provides practical clinical value by enabling nurses to conduct early risk screening and initiate timely, targeted support before mothers leave the hospital.

This study has several strengths. First, in contrast to previous studies on breastfeeding predictors that primarily utilized retrospective designs, this study employed a prospective design and systematically incorporated several key variables related to exclusive breastfeeding. Notably, clinical assessment factors—such as breast comfort, neonatal sucking ability, and nipple type from the LATCH score—were included, enhancing the predictive validity and practical applicability of the model. Second, based on the IMB theoretical framework, this study integrates factors of information, motivation, and behavioral skills, which contributes to understanding the mechanisms underlying breastfeeding behavior from a behavioral science perspective and extends the theoretical foundation for breastfeeding interventions.

This study still has several limitations. First, although it adopted a prospective design and performed external validation in an independent cohort, the validation sample size was relatively limited and derived from the same city, which may restrict the representativeness of the findings and reduce statistical power. Second, differences in maternal demographic characteristics and in-hospital breastfeeding support measures between the validation cohort and the development cohort may have influenced the model’s generalizability, resulting in decreased discrimination in the validation set. Future studies should expand the sample size and conduct multicenter, multi-regional dynamic validation and model updating to enhance the robustness and clinical applicability of the model.

## 5 Conclusion

This study developed a predictive model for exclusive breastfeeding at discharge based on the IMB theory, integrating five key variables: newborn gender, early skin contact, feeding attitude, breastfeeding self-efficacy, and LATCH score. The model is visualized as a nomogram, demonstrating strong predictive performance and clinical applicability. The model aids in early identification of high-risk individuals for feeding and facilitates personalized interventions.

## Data Availability

The original contributions presented in the study are included in the article/Supplementary material, further inquiries can be directed to the corresponding authors.
