# Correction: Construction and validation of a predictive model for exclusive breastfeeding at discharge based on the information-motivation-behavioral skills theory

**DOI:** 10.3389/fmed.2025.1737338

**Published:** 2025-11-14

**Authors:** Na Yin, Shanshan Shan, Jie Bai, Hongxia Lu, Yangyang Wang, Jiaqi Li, Hui Jiang, Ju Zhang

**Affiliations:** 1 College of Nursing, Hangzhou Normal University, Hangzhou, China; 2 Nursing Department, Shanghai First Maternity and Infant Hospital, Obstetrics and Gynecology Hospital Affiliated to Tongji University School of Medicine, Shanghai, China; 3 Affiliated Maternal and Child Health Hospital of East China Normal University, Shanghai Changning District Maternal and Child Health Hospital, Shanghai, China

**Keywords:** information–motivation–behavioral skills (IMB) model, exclusive breastfeeding, risk prediction, nomogram, predictive model

Author Shanshan Shan was erroneously omitted as equal contributing author.

The original version of this article has been updated.

