# Supplementary material for: Construction and validation of a predictive model for exclusive breastfeeding at discharge based on the information-motivation-behavioral skills theory
Source: Front Med (Lausanne). 2025 Oct 30;12:1683293. doi: 10.3389/fmed.2025.1683293 (PMC12611865; doi:10.3389/fmed.2025.1683293)
Supplement: Supplementary file 1 [file Table_1.docx]

Supplementary Material

# Supplementary Data

Table 1: Results of single-factor logistic regression

| Variables | β | S.E | Z | *P* | OR (95%CI) |
| --- | --- | --- | --- | --- | --- |
|  |  |  |  |  |  |
| Education Level |  |  |  |  |  |
| Junior High or Below |  |  |  |  | 1.00 (Reference) |
| High School or Vocational School | -14.47 | 594.16 | -0.02 | 0.981 | 0.00 (0.00 ~ Inf) |
| College or Bachelor's Degree | -14.14 | 594.16 | -0.02 | 0.981 | 0.00 (0.00 ~ Inf) |
| graduate student | -14.22 | 594.16 | -0.02 | 0.981 | 0.00 (0.00 ~ Inf) |
| Household Registration |  |  |  |  |  |
| Local Shanghai |  |  |  |  | 1.00 (Reference) |
| Outside Shanghai | 0.33 | 0.27 | 1.22 | 0.221 | 1.39 (0.82 ~ 2.35) |
| Number of Children |  |  |  |  |  |
| 1 Child |  |  |  |  | 1.00 (Reference) |
| 2 Children | 0.25 | 0.33 | 0.75 | 0.455 | 1.28 (0.67 ~ 2.45) |
| 3 or More Children | 0.43 | 1.09 | 0.39 | 0.693 | 1.54 (0.18 ~ 12.96) |
| Mode of Conception |  |  |  |  |  |
| Natural Conception |  |  |  |  | 1.00 (Reference) |
| Assisted Conception | 0.22 | 0.41 | 0.55 | 0.583 | 1.25 (0.56 ~ 2.78) |
| Pre-delivery BMI |  |  |  |  |  |
| Underweight（＜18.49 kg/m2） |  |  |  |  | 1.00 (Reference) |
| Normal （18.5~24.99 kg/m2） | -0.62 | 0.46 | -1.36 | 0.174 | 0.54 (0.22 ~ 1.32) |
| Overweight （25~29.99 kg/m2） | -0.56 | 0.57 | -0.97 | 0.332 | 0.57 (0.19 ~ 1.76) |
| Obese（≥30 kg/m2） | -0.87 | 0.93 | -0.94 | 0.347 | 0.42 (0.07 ~ 2.57) |
| Mode of Delivery |  |  |  |  |  |
| Vaginal Delivery |  |  |  |  | 1.00 (Reference) |
| Cesarean Delivery | -0.17 | 0.24 | -0.69 | 0.492 | 0.85 (0.53 ~ 1.36) |
| Forceps-assisted Delivery | 0.02 | 0.80 | 0.02 | 0.982 | 1.02 (0.21 ~ 4.88) |
| History of Cesarean Section |  |  |  |  |  |
| None |  |  |  |  | 1.00 (Reference) |
| Yes | -0.47 | 0.41 | -1.14 | 0.255 | 0.63 (0.28 ~ 1.40) |
| Pain Relief Method |  |  |  |  |  |
| Epidural Anesthesia |  |  |  |  | 1.00 (Reference) |
| Spinal Anesthesia | -0.08 | 0.24 | -0.34 | 0.734 | 0.92 (0.57 ~ 1.48) |
| None | 0.78 | 0.76 | 1.02 | 0.310 | 2.17 (0.49 ~ 9.73) |
| Delivery Companion |  |  |  |  |  |
| Yes |  |  |  |  | 1.00 (Reference) |
| No | -0.03 | 0.24 | -0.14 | 0.885 | 0.97 (0.61 ~ 1.54) |
| Newborn Gender |  |  |  |  |  |
| Female |  |  |  |  | 1.00 (Reference) |
| Male | -0.65 | 0.25 | -2.64 | 0.008 | 0.52 (0.32 ~ 0.85) |
| Mother-Infant Separation |  |  |  |  |  |
| No |  |  |  |  | 1.00 (Reference) |
| Yes | 0.15 | 0.29 | 0.51 | 0.611 | 1.16 (0.66 ~ 2.03) |
| Received Breastfeeding Education |  |  |  |  |  |
| Yes |  |  |  |  | 1.00 (Reference) |
| No | 0.22 | 0.30 | 0.72 | 0.472 | 1.24 (0.69 ~ 2.26) |
| Breastfeeding Experience |  |  |  |  |  |
| Yes |  |  |  |  | 1.00 (Reference) |
| No | -0.12 | 0.29 | -0.41 | 0.679 | 0.89 (0.50 ~ 1.57) |
| Perceived Insufficient Milk |  |  |  |  |  |
| Yes |  |  |  |  | 1.00 (Reference) |
| No | 0.12 | 0.25 | 0.49 | 0.623 | 1.13 (0.69 ~ 1.84) |
| Planned Pregnancy and Delivery |  |  |  |  |  |
| Yes |  |  |  |  | 1.00 (Reference) |
| No | -0.18 | 0.35 | -0.51 | 0.611 | 0.84 (0.42 ~ 1.67) |
| First Breastfeeding After Delivery |  |  |  |  |  |
| Within 1 Hour of Delivery |  |  |  |  | 1.00 (Reference) |
| Within 24 Hours of Delivery | 0.01 | 0.34 | 0.02 | 0.981 | 1.01 (0.52 ~ 1.95) |
| After 1 Day of Delivery | -0.68 | 0.55 | -1.23 | 0.218 | 0.51 (0.17 ~ 1.49) |
| Skin-to-Skin Contact |  |  |  |  |  |
| No |  |  |  |  | 1.00 (Reference) |
| Yes | 1.61 | 0.44 | 3.69 | <.001 | 5.01 (2.13 ~ 11.78) |
| Maternity Leave |  |  |  |  |  |
| <3 Months |  |  |  |  | 1.00 (Reference) |
| 3~4 Months | 0.22 | 0.97 | 0.23 | 0.819 | 1.25 (0.19 ~ 8.44) |
| 4~6 Months | 1.42 | 0.72 | 1.98 | 0.048 | 4.15 (1.01 ~ 16.97) |
| ≥6 Months | 1.99 | 0.83 | 2.40 | 0.016 | 7.33 (1.44 ~ 37.33) |
| Family Monthly Income |  |  |  |  |  |
| <5000 RMB |  |  |  |  | 1.00 (Reference) |
| 5000~10000 RMB | -0.02 | 1.13 | -0.02 | 0.984 | 0.98 (0.11 ~ 8.88) |
| >10000 RMB | -0.26 | 1.10 | -0.23 | 0.816 | 0.77 (0.09 ~ 6.73) |
| Breastfeeding Duration Expectation |  |  |  |  |  |
| 1~3 Months |  |  |  |  | 1.00 (Reference) |
| 4~6 Months | 0.66 | 0.44 | 1.51 | 0.131 | 1.93 (0.82 ~ 4.55) |
| 7~12 Months | 0.96 | 0.46 | 2.06 | 0.039 | 2.60 (1.05 ~ 6.44) |
| 13~24 Months | 1.25 | 1.14 | 1.09 | 0.274 | 3.50 (0.37 ~ 32.97) |
| Family Support Level |  |  |  |  |  |
| Low |  |  |  |  | 1.00 (Reference) |
| Medium | -13.89 | 840.27 | -0.02 | 0.987 | 0.00 (0.00 ~ Inf) |
| High | -14.22 | 840.27 | -0.02 | 0.987 | 0.00 (0.00 ~ Inf) |
| Complications |  |  |  |  |  |
| None |  |  |  |  | 1.00 (Reference) |
| Present | -0.17 | 0.28 | -0.62 | 0.535 | 0.84 (0.48 ~ 1.46) |
| Age | 0.01 | 0.03 | 0.35 | 0.729 | 1.01 (0.95 ~ 1.08) |
| BKQ | 0.03 | 0.04 | 0.70 | 0.484 | 1.03 (0.95 ~ 1.11) |
| IIFAS | 0.10 | 0.02 | 4.56 | <.001 | 1.10 (1.06 ~ 1.15) |
| BSES-SF | 0.07 | 0.01 | 4.98 | <.001 | 1.07 (1.04 ~ 1.10) |
| LATCH | 0.40 | 0.10 | 3.98 | <.001 | 1.49 (1.22 ~ 1.81) |

**
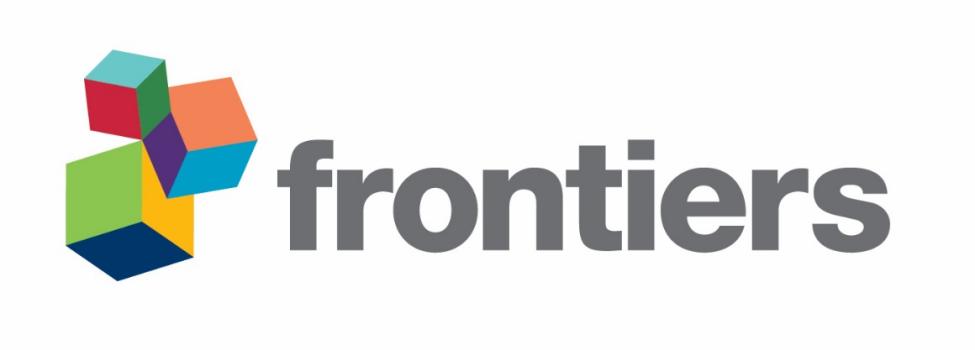
**
